# Awareness of droplet and airborne isolation precautions among dental health professionals during the outbreak of corona virus infection in Riyadh city, Saudi Arabia

**DOI:** 10.4317/jced.52811

**Published:** 2016-10-01

**Authors:** Mohammad-Abdul Baseer, Shahzeb-Hasan Ansari, Sultan-Saleh AlShamrani, Abdul-Rahman Alakras, Raif Mahrous, Abdul-Majeed Alenazi

**Affiliations:** 1 Assistant Professor, Department of Preventive Dentistry, Riyadh Colleges of Dentistry And Pharmacy, An-namuthajiya Campus, P. O Box: 84891, Riyadh 11681, Kingdom of Saudi Arabia; 2 Lecturer, Department of Preventive Dentistry, Riyadh Colleges Of Dentistry And Pharmacy, An-namuthajiya Campus, P. O Box: 84891, Riyadh 11681, Kingdom of Saudi Arabia; 3 Post-Graduate student, Department of Restorative Dentistry, An-namuthajiya Campus, P. O Box: 84891, Riyadh 11681, Kingdom of Saudi Arabia; 4 General Dental practitioner, An-namuthajiya Campus, P. O Box: 84891, Riyadh 11681, Kingdom of Saudi Arabia; 5 General Dental practitioner, Department of Restorative Dentistry, An-namuthajiya Campus, P. O Box: 84891, Riyadh 11681, Kingdom of Saudi Arabia

## Abstract

**Background:**

This study aimed to determine knowledge, attitude and practice of airborne and droplet isolation precautions among Dental Health Professionals (DHPs) (dental students, interns, practitioners and auxiliaries) during the outbreak of MERS (Middle East Respiratory Syndrome), corona virus infection in Riyadh city, Saudi Arabia.

**Material and Methods:**

A cross-sectional survey was conducted among 406 dental health professionals (DHPs) working in selected dental facilities in Riyadh city, Saudi Arabia during the outbreak of MERS (April-June 2013). A structured, close-ended, self-administered questionnaire explored the knowledge, attitude, and practice towards droplet and isolation precautions. Collected data was subjected to descriptive statistics to express demographic information, mean knowledge score, mean attitude score and practice score of DHPs. Inferential statistics (Mann-Whitney U test and Kruskal Wallis tests, p < 0.05) were used to examine differences between study variables. Spearman’s rho correlation was used to identify the association between the knowledge-attitude, knowledge-practice, and attitude-practice.

**Results:**

A response rate of rate of 90.22% (406 out of 452) was obtained. The mean scores of knowledge, attitude and practice were 10.61 ± 1.19, 50.54 ± 7.53 and 8.50 ± 2.14 respectively. Spearman’s correlation test revealed a significant linear positive correlation between knowledge and attitude (r-0.501, *P*- 0.01), knowledge and practice (r-0.185, *P*-0.01) and attitude and practice (r-0.351, *P*- 0.01) of DHPs about airborne isolation precautions.

**Conclusions:**

Dental health professionals considered in the present study showed good knowledge, positive attitude and good practice towards droplet and airborne isolation precautions during outbreak of MERS.

** Key words:**Knowledge, attitude, practice, droplet, airborne, precaution, dental professionals.

## Introduction

Middle East Respiratory Syndrome (MERS) is a viral respiratory disease caused by corona virus called Middle East Respiratory Syndrome – Corona virus (MERS-CoV). This is a novel virus belonging to genus Beta coronavirus and was first reported in Kingdom of Saudi Arabia (KSA) in September 2012 (1). Middle East respiratory syndrome coronavirus (MERS-CoV) summary and literature update-as of 9 May 2014 in KSA suggested that more than 60% patients including health care workers have acquired infection in hospital setting. Besides, 15% of health care workers tested positive with MERS-CoV severe infection were either admitted to intensive care unit or died. Last update (7 March 2015) by European Centre for Disease Prevention and Control on MERS reported a total of 938 cases and 402 deaths in KSA, suggesting epicenter of outbreak, with health care workers at highest risk of acquiring infection. Mode of transmission was mainly by close contacts with infected person such as those caring for or living with them. However, virus transmission through a hospital cluster suggested that the mode of spread is through contact and in the form of droplets (2).

With reported morbidity and mortality of health care professionals contracted with MERS-CoV has pointed out inadequate infection control practices in health care settings. This led Ministry of Health to re-emphasize the need for strict compliance for infection control guidelines prescribed by World Health Organization (WHO) and Center for Disease Control and prevention (CDC) in health care facilities by creating awareness among health care workers. These guidelines encompassed standard precautions, droplet and airborne precautions and eye protection while dealing with suspected or infected cases of MERS-CoV (3). Recent study conducted among healthcare workers from KSA showed a good knowledge and positive attitude towards MERS (4).

Dental clinic is a common health care facility in which aerosol (airborne/droplet) particles are produced during various treatment procedures. These treatment procedures include; scaling by ultrasonic scaler, air polishing, tooth preparation with a high and low speed rotary instruments, use of air-water syringe and air abrasion etc. The aerosol thus produced is a mixture of particles and fluid containing pathogenic virus. The production of airborne material during dental procedures is apparent to the dentist, the dental team and the patient (5).

Previous studies have reported that the dental health professionals (DHPs) and patients are at high risk of infections from different types of bacteria (Mycobacterium Tuberculosis, Staphylococci) virus (Hepatitis B and C virus, herpes simplex virus types 1 and 2 and Human Immunodeficiency virus) and fungus (6,7). Additionally occurrence of asymptomatic and subclinical MERS-CoV cases in population could pose a huge threat to dental practice by transmitting infections between dentist and patients and the dental team. To ensure safe working environment and to prevent transmission of infection in dental practice CDC developed guidelines, which mainly included standard precaution and transmission based isolation precautions (airborne, droplet and contact precautions). Strict adherences of these guidelines are needed to prevent the potential spread of infection in dental practice.

To the best of our understanding, none of the previous studies examined general awareness of droplet and airborne isolation precautions among the DHPs from Saudi Arabia, especially when the concerns of infection control among health care workers increased due to the outbreak of MERS-CoV infection (April-May-June 2013). Hence the aim of this study was to determine knowledge, attitude and practice of airborne and droplet isolation precautions among DHPs (dental students, interns, practitioner and auxiliaries) in Riyadh city, Saudi Arabia.

## Material and Methods

-Study design and participants

A cross-sectional survey was conducted for three months from April-June 2013, in various dental care facilities in Riyadh City, Saudi Arabia. Sampling was carried out in two stages- first stage list of dental care facilities (Private, government military and university) in Riyadh city was prepared, second stage DHPs working in these facilities were then selected into the study. List of private hospitals and polyclinics providing dental care in Riyadh city was obtained by using health insurance provider network, and the list of universities providing dental care was obtained by visiting ministry of higher education website of Saudi Arabia. Similarly, list of government armed forces dental care facilities in Riyadh city was also prepared. From among the above mentioned dental care facilities few dental centers were selected randomly by applying lottery method. DHPs including clinical level dental students, interns, dental practitioners and dental auxiliaries working in these facilities were invited to participate in the study. A convenience sampling methodology was utilized to recruit DHPs based on ease of availability during survey period. Trained dental interns approached DHPs in their work place and distributed the questionnaires along with required instructions. Confidentiality of the data was assured to DHPs. All the participants were informed about the purpose and scope of the study and those who agreed to sign the consent form were considered in this study.

Sample size was determined by using G* power statistical power analysis program 3.1.1(8). A sample size of 452 was determined by considering an alpha of 0.05, a power of 0.85, and effect size of *ρ* = 0.14 for a two-tailed Spearman correlation test

-Questionnaire design: Instrument developed by Askerian *et al.* (9) and Jain *et al.* (10) in line with the CDC guidelines for evaluating awareness of droplet and airborne isolation precautions among dental students and faculty was slightly modified and validated in two steps. Firstly, the study instrument was sent to professionals from public health and dental public health background to give their expert opinion with regards to its ease, relativity and importance. Secondly, pretesting of the questionnaire was carried out by choosing a small sample of DHPs (n=20), who provided their views on making the questionnaire simpler and shorter. Amendments from the participants were considered and incorporated into the questionnaire. After in-depth discussion, questionnaire was finalized by the authors and subsequently a pilot study was conducted on a sample of 50 DHPs by using modified version of the questionnaire to ensuring comprehensibility and reliability. A Cronbach’s alpha of 0.80 was obtained which was deemed satisfactory for conducting this study.

Questionnaire was divided into four parts. The first part included demographic information of the respondents. Second, third and fourth parts evaluated Knowledge, attitude and practice of DHPs regarding the droplet and airborne isolation precautions by eliciting responses on 11 questions in each section. The knowledge was assessed at three different levels (yes, no, I do not know), and a score of 1 was allocated when the answers to questions were in agreement with the CDC guidelines. Therefore, the score for knowledge ranged between 0 (all wrong answers) to 11 (all correct answers). A cut off level of <7 were set for poor knowledge and ≥7 for good knowledge. Attitude was assessed at three possible levels (very strong, strong and null). The responses very strong and strong were assigned a score of 5 and null response was scored at 1 point. Thus, the aggregate score ranged between 11(all null score) to 55 (all questions responded as strong or very strong). A cut off level of <35 was set for negative attitude and ≥35 for positive attitude. In practice section, four levels of responses (always, often, sometimes and never) were utilized by assigning score of 1 for correct answer and a score of 0 for all other responses. Hence the total practice score ranged 0 to 11. Similarly, A cut off level of <7 were set for poor practice level and ≥7 for good practice.

Ethical clearance: Research center of Riyadh Colleges of dentistry and pharmacy formally approved the study.

-Statistical Analysis

Normality distribution of the data was checked by applying Kolmogorov-Smirnov and Shapiro-Wilks tests. Both these tests showed significant *p* value (*p*<0.05) indicating non-normal distribution of the data. Hence non-parametric tests for inferential statistics were applied.

Statistical analysis of the data was carried out by using SPSS version 21. Descriptive statistics was performed and data reported as percentage and frequency. Means and standard deviations for the knowledge, attitude and practice scores were computed. Inferential statistics of Mann-Whitney U test and Kruskal Wallis tests were applied to study variables. A *p* value of <0.05 was considered significant. Spearman’s correlation coefficient was used to compute the correlation between knowledge-attitude, knowledge-practice and attitude-practice. A two tailed *p* value of <0.05 was considered significant for all statistical purposes.

## Results

A total of 406 DHPs responded to the questionnaire giving the response rate of 90.22% (406 out of 452). Majority of them were male (59.9 %) and belonged to all dental health professional categories with undergraduates/graduates most in number (89.4%). Nearly half of the study participants mainly worked in university dental clinics with majority (57.1%) of them having 1-2 years of experience, and (57.1%) of them were Saudi nationals. The characteristics of respondents are mentioned in  Table 1.

**Table 1:**
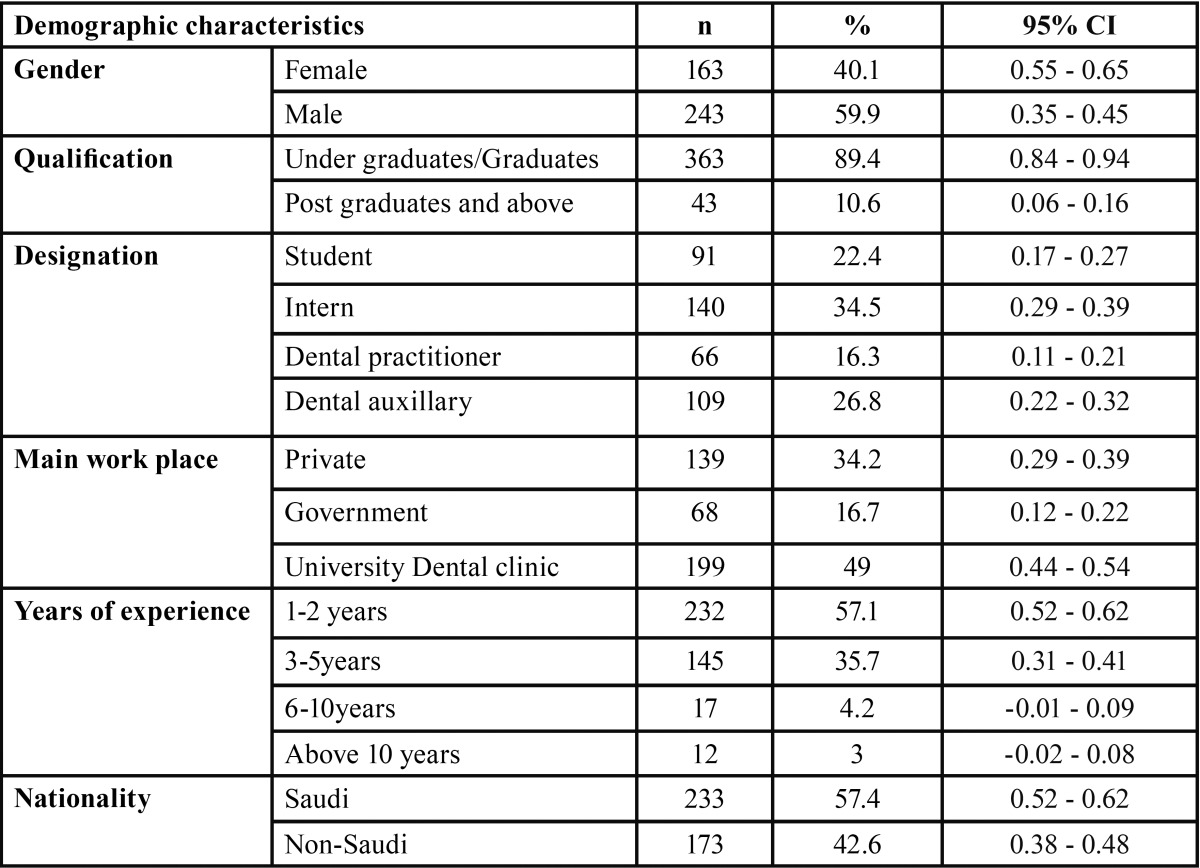
Distribution of Dental health professionals according to their characteristics.

 Table 2 shows the frequency distribution of responses of the study participants to the knowledge, attitude and practice on droplet and airborne isolation precautions. The results of the study revealed that for knowledge the percentage of correct answers was lowest (56.4%) for the question number 4 (Mask should be worn if or when a subject is within a 90 cm distance from a patient under droplet precaution care) and was highest (87.9%) for the question number 1 (Patients with a droplet spread disease should be isolated in a private room). This suggests that the DHPs are very well knowledgeable of the droplet spread diseases and isolation of patients with such diseases. Similarly, for attitude towards airborne and droplet precautions lowest and highest responses were observed for the question numbers 10 (Wards should be notified prior to receiving a patient requiring airborne precautions) and 5 (Hospital wards should be notified prior to receiving a patient needing droplet precautions) respectively. Additionally, frequency of correct response for practice section was lowest for the 2nd, 6th and 11th questions and highest for the question number 3 (Patients with a droplet spread disease should wear a mask during transport).  Table 3 shows the mean and standard deviation of knowledge, attitude and practice scores for different groups. A total mean score for knowledge was 10.61 ± 1.19; Mean scores for attitude and practice 50.54 ± 7.53 and 8.50 ± 2.14 were obtained respectively.

**Table 2:**
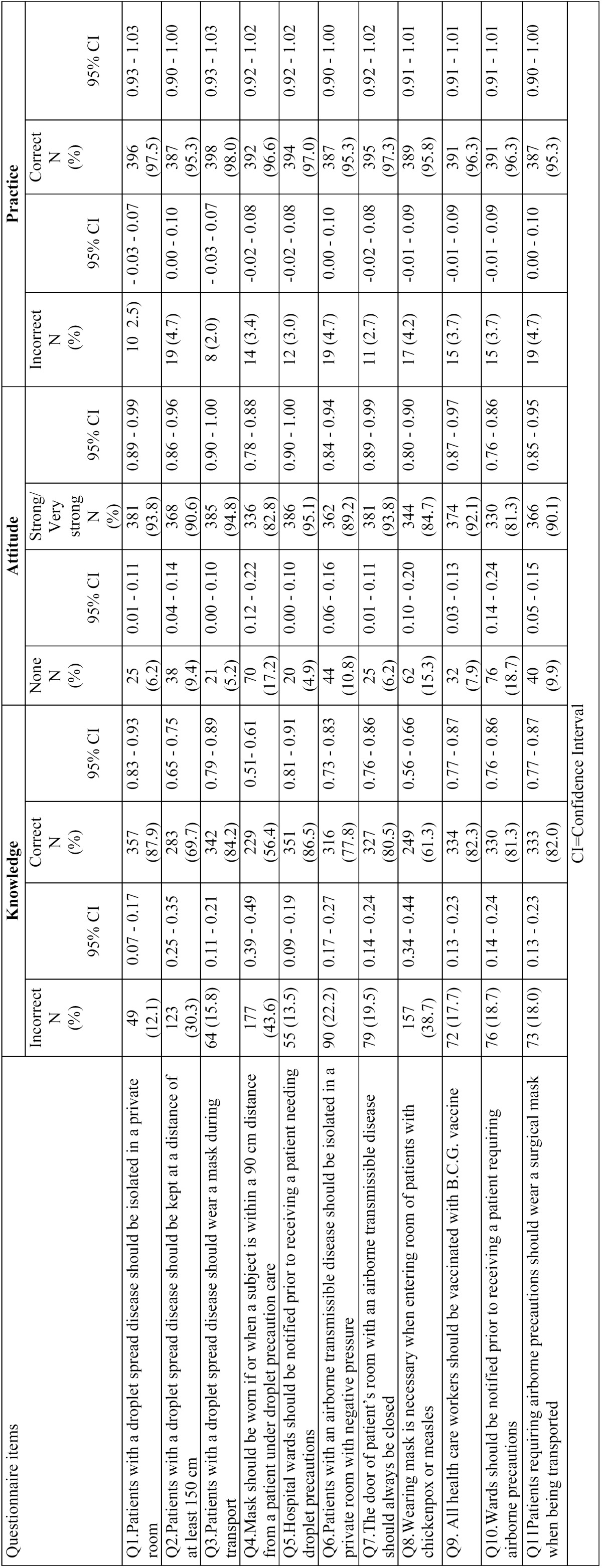
Frequency distribution of answers about the knowledge, attitude and practices on droplet and airborne precautions.

**Table 3:**
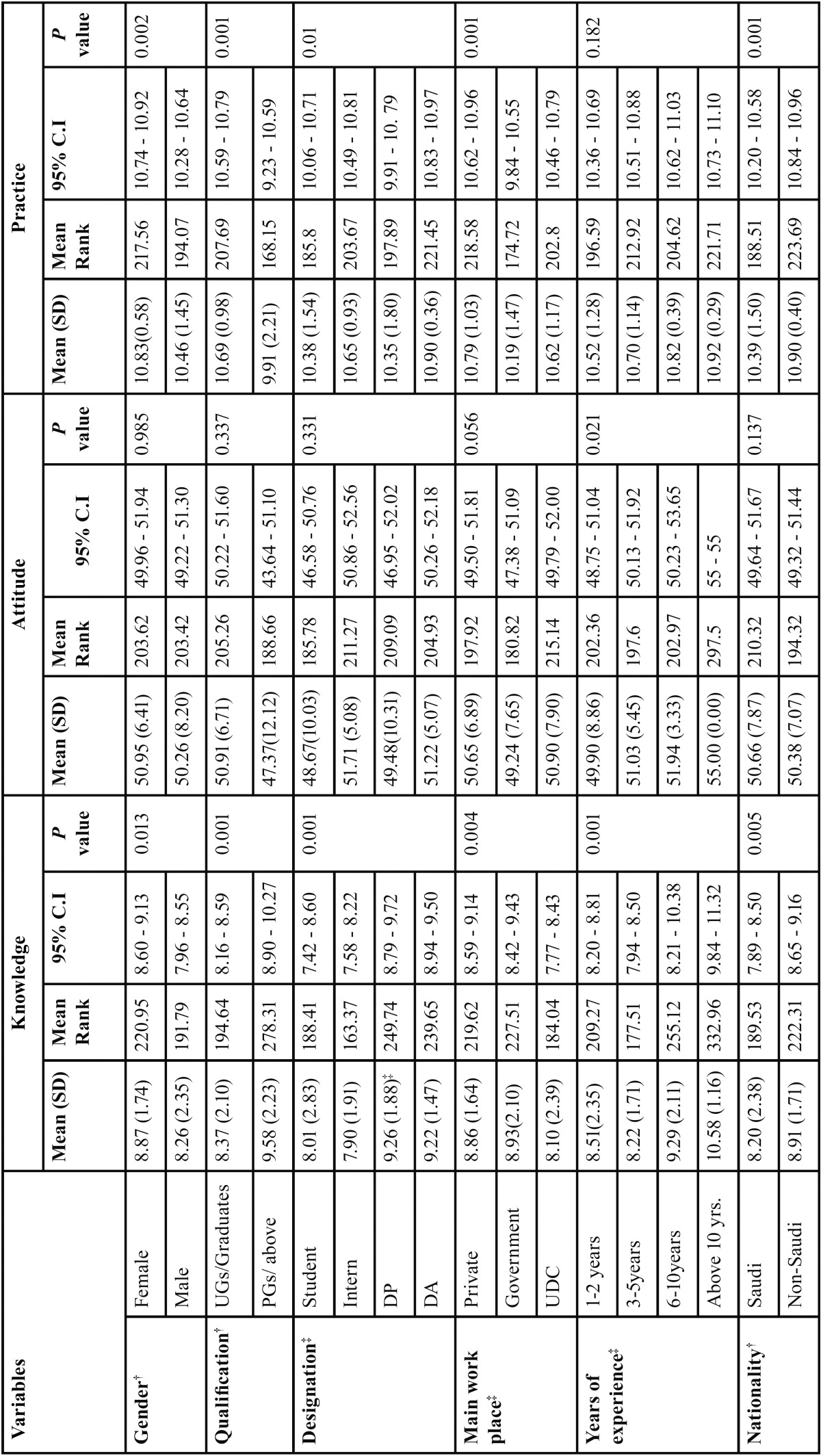
Mean and Standard deviations of knowledge, attitude and practice scores.

All the demographic variables (gender, qualification, designation, main work place and nationality) were significantly associated with mean knowledge score *p*<0.05. Female (8.87 vs 8.21, *p* = 0.013), those having Post graduate and above qualification (9.58 vs 8.37, *p*=001) and non-saudi (8.91 vs 8.20, *p*=005) DHPs showed significantly higher knowledge score as compared to their counter parts. Dental practitioners, those working in government sector and having experience of above 10 years showed significantly (*p*<0.05) high mean knowledge score. On contrary years of experience was only the demographic variable significantly associated with mean attitude score. DHPs with above 10 years of experience showed significantly (*p*<0.05) high attitude score within the group. Similarly, all the demographic variables were significantly associated with practice score except for the years of experience.

Kruskal-Wallis tests with Bonferroni-Holm corrected Mann-Whitney post hoc tests for multiple comparisons for type of DHPs showed statistically significant differences (*p*<0.01) in knowledge between dental students and dental practitioners, students and dental assistants, interns and dental practitioners, and interns and dental assistants. However attitude did not differ significantly among DHPs. But Practice score significantly differed between dental students and dental assistants (*p*<0.01). Similarly, DHPs working in the private sector showed significantly high mean rank compared to the government DHPs (*p*<0.01).

Spearman’s correlation test revealed a significant linear positive correlation between knowledge and attitude(r-0.501, *P*- 0.01), knowledge and practice (r-0.185, *P*-0.01) and attitude and practice (r-0.351, *P*- 0.01) of DHPs about airborne isolation precautions  Table 4. In general study results suggested that DHPs considered in the present study showed a good knowledge, positive attitude and good practice towards droplet and airborne isolation precautions.

**Table 4:**
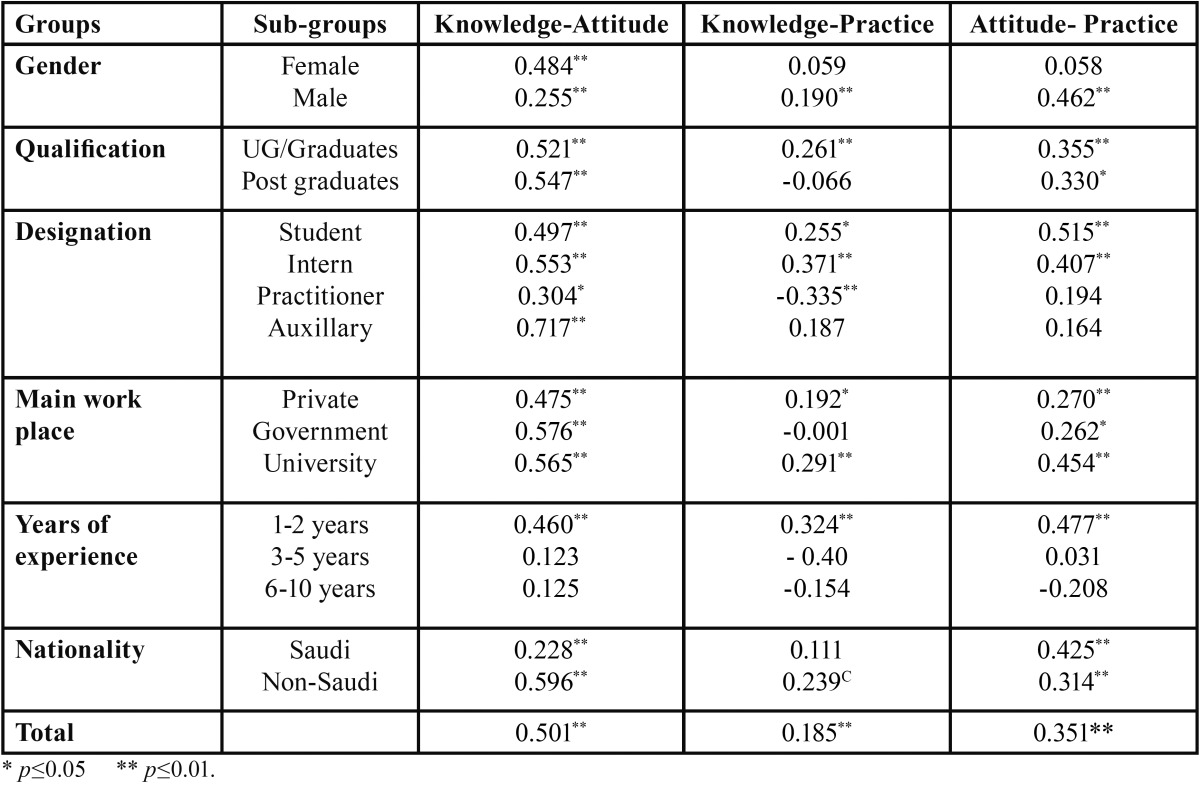
Spearman’s correlation coefficients between knowledge- attitude (K-A), knowledge - practice (K-P) and attitude - practice (A-P) practice scores regarding droplet and airborne precautions.

## Discussion

To the best of our understanding, there were only two previous reports of similar studies, particularly none that examined the DHPs knowledge, attitude and practices towards droplet and airborne isolation precautions from Saudi Arabia, especially during the outbreak of MERS. In view of this limited publications, the comparison of our findings has been made with other related conditions such as infection control compliance among dental health professionals. Overall, DHPs considered in the present study exhibited good knowledge, positive attitude and good practice of droplet and isolation precautions.

Previous studies have reported a mean knowledge score of 6.71± 0.99, 9.17±2.07 (9,10) while in the present study mean knowledge score of 10.61±1.19 was observed among DHP. This discrepancy in the knowledge could be explained by the fact that the present study was conducted during the outbreak of MERS and the referenced studies were carried out in the year 2005 and 2010, since those time lots of advancements have been made in the information-technology in spread of health alerts. There has been increase in the internet access and usage among dental professionals to receive information about infection control (11). Moreover, now a day’s internet is widely available and utilized to gain knowledge on evolving disease by many health care professionals including DHPs (12). Educational materials posted on the website of the ministry of health during the outbreak of MERS CoV could be one of the major sources of knowledge among health professionals including DHPs. Moreover, individualized text messages sent by the relevant professional agencies to their registered health professionals could be the source of this knowledge. In addition, seminar, symposium and research articles could be the source of knowledge about isolation precautions among dental health professionals during the MERS outbreak (4). All above mentioned factors could have played role in increasing knowledge of isolation precautions among DHPs. Even though some knowledge gaps have been identified in the present study such as; distance at which mask should be worn while approaching the patient under droplet precautionary care and wearing mask while entering room of patients with chickenpox or measles requiring further information.

A very high percentage (87.9%) of respondents knew that the patients with a droplet spread disease should be isolated in a private room. One could speculate that the recent outbreak of MERS in Saudi Arabia has led the relevant authorities to initiate educational campaign targeted towards public and health professionals. These campaigns mainly focused on prevention and symptoms of MERS (4). Dental practitioners and DHPs having above 10 years of experience showed high level of knowledge towards isolation precautions. This could be due to the in depth theoretical courses received and experience gained over a period of time in infection control practices.

Previous studies have reported a mean attitude scores of (10.51± 6.26) and (48.65± 7.47) respectively (9,10). However, present study showed a higher mean attitude score of (50.54±7.53). Except for the years of experience none of the demographic variables showed significant difference in the mean attitude score. DHPs with above 10 years of experience had more positive attitude as compared to other less experienced DHPs. This suggests that as the experience of DHPs increases their attitude towards isolation precautions become more positive.

Lowest response was observed for the question Wards should be notified prior to receiving a patient requiring airborne precautions indicating room for improvement.

In Practice section, all the responses to the questions were found to be above 95% with the mean practice score of 8.50±2.14. This indicates good practice towards isolation precaution measure prescribed by CDC. However, previous studies had shown a mean practice score of 2.68± 3.16 and 6.88±3.51 respectively (9,10). High practice mean score was found among DHPs with above 10 years of experience.

In the present study high percentage of correct responses and significant positive correlations were observed between knowledge-attitude, knowledge-practice and attitude-practice suggesting good knowledge and compliance towards droplet and isolation precautions among DHPs, as recommended by CDC. This high level of correlation could be due to the recent educational campaigns targeted towards MERS prevention programs by relevant authorities in KSA. Thus it can be speculated that continuous exposure of DHPs to the various educational programs which emphasize the importance of implementing CDC guidelines are more likely to use information gathered in their practice (13). Additionally, DHPs with more positive attitudes are motivated to explore more information to increase their understanding of isolation precautions. The reason for such correlation could be described by the theory of Reasoned Action, which states that the individual’s intention to a specific behaviour is a function of their attitude towards that behaviour (14).

Study results are in line with that reported by Jain *et al.*, in which positive correlation between knowledge-attitude, knowledge-practice and attitude-practice and fair compliance towards infection control guidelines was observed (10). Conversely, studies have also reported acceptable knowledge, and attitude with poor compliance towards isolation precautions among DHPs (9,15).

Despite several recommendations and guidelines issued by national and international medical and dental societies and governmental agencies studies have shown inadequate infection control in dental and medical care facilities (16). Previous studies have reported lack of compliance towards adherence to the measures of infection control among DHPs (17-20). Several barriers to compliance towards standard precautions have been identified such as; lack of knowledge and technical difficulties, inadequate facilities, heavy workload, patient expectations, inter-professional conflicts, and lack of good role models, financial issues and unsupportive organizational culture (21). It has been reported that most of DHPs show compliance towards infection control practices as per their needs but no necessarily according to the real recommendations (22).

This is the first study to report the level of knowledge, attitude and practices of DHPs toward airborne and droplet isolation precautions during the MERS outbreak in Saudi Arabia. It has highlighted the area where very little research has been done in identify knowledge gaps towards isolation precautions. In spite of good study findings we admit some limitations, and cautions must be taken while generalizing the results of the study due to the convenient sampling methodology, limited sample size with some reduced response rate, sample clustering from single city, and statistical errors due to multiple significance tests.

In general, dental health professionals considered in the present study showed good knowledge, positive attitude and good practice towards droplet and airborne isolation precautions during outbreak of MERS. However there is still scope for improvements towards droplet and airborne isolation precautions among DHPs. Extensive educational campaigns are needed to fill the existing lacunae in the knowledge, attitude and practices. It is highly desirable for relevant dental professional organizations to emphasize occupational and educational campaigns to increase awareness towards isolation precautions among DHPs.
